# Application of Mycorrhiza and Soil from a Permaculture System Improved Phosphorus Acquisition in Naranjilla

**DOI:** 10.3389/fpls.2017.01263

**Published:** 2017-07-19

**Authors:** Sarah Symanczik, Michelle Gisler, Cécile Thonar, Klaus Schlaeppi, Marcel Van der Heijden, Ansgar Kahmen, Thomas Boller, Paul Mäder

**Affiliations:** ^1^Department of Soil Sciences, Research Institute of Organic Agriculture Frick, Switzerland; ^2^Department of Environmental Sciences, University of Basel Basel, Switzerland; ^3^Department of Agroecology and Environment, Agroscope Zürich, Switzerland

**Keywords:** naranjilla, arbuscular mycorrhizal fungi, fungal communities, *Piriformospora indica*, farming practices, permaculture, next generation sequencing

## Abstract

Naranjilla (*Solanum quitoense*) is a perennial shrub plant mainly cultivated in Ecuador, Colombia, and Central America where it represents an important cash crop. Current cultivation practices not only cause deforestation and large-scale soil degradation but also make plants highly susceptible to pests and diseases. The use of arbuscular mycorrhizal fungi (AMF) can offer a possibility to overcome these problems. AMF can act beneficially in various ways, for example by improving plant nutrition and growth, water relations, soil structure and stability and protection against biotic and abiotic stresses. In this study, the impact of AMF inoculation on growth and nutrition parameters of naranjilla has been assessed. For inoculation three European reference AMF strains (*Rhizoglomus irregulare*, *Claroideoglomus claroideum*, and *Cetraspora helvetica*) and soils originating from three differently managed naranjilla plantations in Ecuador (conventional, organic, and permaculture) have been used. This allowed for a comparison of the performance of exotic AMF strains (reference strains) versus native consortia contained in the three soils used as inocula. To study fungal communities present in the three soils, trap cultures have been established using naranjilla as host plant. The community structures of AMF and other fungi inhabiting the roots of trap cultured naranjilla were assessed using next generation sequencing (NGS) methods. The growth response experiment has shown that two of the three reference AMF strains, a mixture of the three and soil from a permaculture site led to significantly better acquisition of phosphorus (up to 104%) compared to uninoculated controls. These results suggest that the use of AMF strains and local soils as inoculants represent a valid approach to improve nutrient uptake efficiency of naranjilla and consequently to reduce inputs of mineral fertilizers in the cultivation process. Improved phosphorus acquisition after inoculation with permaculture soil might have been caused by a higher abundance of AMF and the presence of *Piriformospora indica* as revealed by NGS. A higher frequency of AMF and enhanced root colonization rates in the trap cultures supplemented with permaculture soil highlight the importance of diverse agricultural systems for soil quality and crop production.

## Introduction

Naranjilla (*Solanum quitoense*, Solanaceae) is a perennial shrub and an important cash crop in Ecuador, Colombia, and Central America where its fruits are used for a very popular beverage (Acosta et al., 2009). However, the cultivation of naranjilla causes severe ecological problems. Often located at steep sites and cultivated in monoculture, plantations are fully exposed to erosion and soil degradation (Revelo et al., 2010). In the course of this study, highly different measurements were obtained when comparing the soil quality of a conventional 2 years old naranjilla plantation in La Esperanza (province Carchi, Ecuador) and native forest soil directly adjacent to the plantation. Indeed, an enormous loss of soil organic matter (SOM, -66%) and available nutrients such as phosphorus (P, -85%), nitrogen (N, -52%), magnesium (Mg, -70%), potassium (K, -30%), and calcium (Ca, -55%) was detected after only 2 years of cultivation (Supplementary Table S1). Similarly, Mainville et al. (2006) have shown that deforestation dramatically reduced soil fertility in the Andean Amazon. Due to the rapid loss in soil nutrients, high inputs of mineral fertilizers are regularly applied to these plantations. Furthermore, naranjilla plants suffer from several diseases and pests, the most important ones being fusarium wilt, late blight, nematode (*Meloidogyne incognita*), and fruit borer (*Neoleucinodes elegantalis*) infestations (Revelo et al., 2010). In order to fight these pests an array of different pesticides is used by the farmers. However, after 2–3 years the productivity of the plantation decreases due to erosion, lack of nutrients and the high prevalence of resistant pathogens in the soil (Revelo et al., 2010). As a consequence, the land gets abandoned or converted into pasture and a new piece of primary or secondary forest is chosen for a new naranjilla plantation (Heiser, 1985; Revelo et al., 2010). Thus, the cultivation of naranjilla poses serious ecological problems relating to deforestation and forest soil degradation (Sowell and Shively, 2012) within some of the world’s richest biodiversity hotspots as the Northern Ecuadorian mountain cloud forests (Brummitt and Lughadha, 2003).

Breeding of resistant varieties and grafting on resistant root stocks are current practices to improve pest resistance of naranjilla (Sowell and Shively, 2012). However, the problem of soil erosion and soil degradation remains unsolved. To counteract the negative impacts of current cultivation practices resilient and sustainable alternatives are crucially needed. Organic farming and permaculture systems pose valuable options. Both systems are known to widely exclude chemical fertilizers and pesticides and to apply the principle of diversification in terms of crop rotation, inter or mixed cropping to promote and maintain soil fertility (Mäder et al., 2000).

It has been shown that arbuscular mycorrhizal fungi (AMF) which form associations with 80–90% of all terrestrial plants (Parniske, 2008) can contribute to alleviate problems related to intensive cultivation practices. AMF were shown to fulfill a broad spectrum of beneficial functions for their host plants as well as for their environments like improved nutrient and water uptake, enhanced tolerance against biotic and abiotic stresses and improved soil structure to counteract soil erosion (Azcón-Aguilar and Barea, 1997; Augé, 2001; Hildebrandt et al., 2007; Pozo et al., 2010; Smith and Read, 2010; Porcel et al., 2012; Leifheit et al., 2014). At the same time it has been observed that intensive agricultural practices like the use of large amounts of mineral fertilizers and pesticides can have severe effects on AMF and other fungal symbionts (Bünemann et al., 2006; Kalia and Gosal, 2011) and consequently can negatively impact plant nutrition and growth (Verbruggen et al., 2010). Indeed it has been shown that the loss of fungal diversity disrupts major ecosystem services such as ecosystem variability and productivity and might decrease plant biodiversity (Van der Heijden et al., 1998; Wagg et al., 2014). On the other hand, there are several agricultural practices that can stimulate the presence of beneficial soil biota, such as crop rotation, diversification or reduction of tillage intensity (Köhl et al., 2014). However, there are no data available comparing the impact of different agricultural management practices such as conventional versus organic and permaculture systems on fungal communities in tropical rain forest ecosystems.

Beside the wide range of beneficial properties, AMF can be used as inoculant and act as a biological fertilizer and disease control agent in naranjilla problematics. Up to date, there are only few studies investigating the association of naranjilla and AMF. Collazos et al. (1986) analyzed the combined effect of inoculation of naranjilla with 10 different AMF species and P fertilization comparing three P levels (0, 50, and 100 kg P/ha). The authors observed increased leaf areas and highest P uptake after inoculation with *Acaulospora* sp., *Glomus* sp., and *Entrophospora colombiana* when grown without P and with the addition of 50 kg P/ha. Also Gonzalez and Osorio (2015) observed that inoculation with *Glomus aggregatum* increased biomass of naranjilla especially when grown under low P levels. Similarly Casierra-Posada et al. (2013) observed greatest biomass production after inoculation with *Scutellospora heterogama* and a mix of *Glomus* spp.

The current study aimed at testing a novel approach by using local soil from three different types of naranjilla plantations for inoculation in addition to three European reference AMF strains commonly used in inoculation experiments. A growth response experiment tested the impact of inoculation on biomass production and nutrient uptake of naranjilla at two different sampling times. In a second experiment, the same local soils were used to establish trap cultures enabling the study and description of native fungal communities in particular AMF inhabiting the roots of naranjilla using a next generation sequencing (NGS) approach. This description had the objective to reveal potential differences in the AMF community structure as affected by agricultural management practices of naranjilla plantations.

## Materials and Methods

### Biological Material

#### Plant Material

Seeds of naranjilla (*Solanum quitoense*, cv. INIAP QUITOENSE 2009) have been obtained from Palora, a canton situated in the province of Moròna Santiago in the eastern part of Ecuador (lat: -1,745709°, long: -77,91837°) and were germinated in AMF-free potting substrate (Ökohum Anzuchterde, Herrenhof, Switzerland) within seed trays at 20°C, 80% relative humidity and 8 h light regime in a glasshouse in the Botanical Garden of the University of Basel, Switzerland. After 21 days seedlings were transplanted and grown for another 21 days in compartmented seed trays with the same substrate. Uniform naranjilla seedlings at five leaf stage were used to start the growth response experiment and to initiate the trap cultures.

#### Inoculum Preparation

For inoculation, three well-studied, “reference AMF species” and a mix of the same three species (AMF mix) were used: *Rhizoglomus irregulare* (BEG 75, 15 spores/g inocula), previously referred to as *Rhizophagus irregularis* and earlier as *Glomus intraradices*, *Claroideoglomus claroideum* (BEG 155, 200 spores/g inocula), and *Cetraspora helvetica* (BEG 153, 13 spores/g inocula), previously referred to as *Scutellospora pellucida*. *R. irregulare* was produced in pot cultures with *Hieracium pilosella, Plantago lanceolata*, and *Allium porrum* as host plants in the greenhouse of the Botanical Institute, Basel, Switzerland, whereas *Cl. claroideum* and *Ce. helvetica* were produced in pot cultures with *P. lanceolata* and *A. porrum* as host plants in the greenhouse of the research station of ETH Zürich, Lindau-Eschikon, Switzerland.

The second type of inoculum were local soils (classified as andosols) coming from three directly adjacent but differently managed naranjilla plantations in Guamaní, in the canton Archidona in the province of Napo in Eastern Ecuador. The first sample was taken from a conventionally managed naranjilla plantation (Conv soil), the second from an ecologically managed plantation (Org soil) and the third from a permaculture site (Perm soil). Details about management practices and soil parameters of the three soils are given in **Table 1** and Supplementary Table S1. Soil parameters were analyzed by lbu (Labor für Boden- und Umweltanalytik, Eric Schweizer AG, Thun, Switzerland). For each site, an area of 25 m × 25 m was chosen from which soil samples of four random subplots of 5 m × 5 m were collected. Each subplot sample consisted of 10 subsamples of individually collected, pooled and sieved (5 mm) naranjilla rhizosphere soil at a depth of 5–20 cm. For the growth response experiment, all four subplot soil samples were pooled to obtain one local soil inoculum per site. For trap culture establishment subplot samples were used independently (an overview scheme is given in Supplementary Figure S1).

**Table 1:** Overview of the three local soils collected from differentially managed naranjilla plantation in Guamani (province Napo, Ecuador) used as local soil inoculants.

	Conventional soil	Organic soil	Permaculture soil
Location	lat: -0.706462° long: -77,610998°	lat: -0.721087° long: -77,608609°	lat: -0.720108° long: -77,613457°
System	Conventional	Organic	Organic
Fertilization	NPK fertilizer (ratio 10:30:10)	Worm and green waste compost, molasses	Worm and green waste compost, chicken manure
Pesticides	Ridomil, Cypermethrin, 2,4-Dichlorophenoxy-acetic acid	–	–
Age of plantation	1 year	1½ years	8 years
Cultivated plants	Naranjilla in monoculture	Naranjilla in monoculture (with sparsely scattered plants as banana, yucca, guavas, maize, dragon fruit, pineapple, peanuts)	Naranjilla amongst wild plants and banana, yucca, guavas, maize, dragon fruit, pineapple, peanuts
**Soil parameters**		
pH (H_2_0)	5.4	5.4	6
P_Olsen_ (mg/kg)	38.1	45.7	47
P^∗^ (mg/kg)	3.6	3.2	4.7
K^∗^ (mg/kg)	43.7	115	195.2
Mg^∗^(mg/kg)	17.4	52.6	192.3
Ca^∗^ (mg/kg)	227	383	1626
N_tot_ (g/kg)	6.9	9.6	12.3
N_min_ (mg/kg)	72.4	82.3	176.8
C_org_ (%)	9.7	15.6	20.3

*^∗^1:10 EDTA extraction.*

*Conv soil, conventionally managed soil; Org soil, organically managed soil; Perm soil, soil from a permaculture system; NPK fertilizer, nitrogen-phosphorus-potassium fertilizer. Soil parameters were analyzed by lbu (Labor für Boden- und Umweltanalytik, Eric Schweizer AG, Thun). For complete report see Supplementary Table S1.*

### Experimental Design

#### Growth Response Experiment

Uniform naranjilla seedlings were individually transplanted into 440 ml plastic pots (gvz-rossat, Otelfingen, Switzerland) filled with 320 g experimental substrate consisting of heat-sterilized (160°C, 3 h) sand (quartz sand, 0.125–0.25 mm; Kaltenhouse, Alsace, France), Vermiculite [Vermiculit Typ “SF” (0.4–1.2 mm), gvz-rossat, Otelfingen, Switzerland] and autoclaved (120°C, 20 min) topsoil (from a local site in Buus, Switzerland) in the ratio 1:1:2 (v:v:v). After 14 weeks, half of the plants including the substrate were transferred into 1 L pots (gvz-rossat, Otelfingen, Switzerland) filled with additional 541 g of the experimental substrate. Nine treatments were applied: four reference AMF treatments with *R. irregulare*, *Cl. claroideum*, *Ce. helvetica* and a mix of the three AMF species (AMF mix); three local soil treatments, namely Conv soil, Org soil, and Perm soil; and two control treatments comprising an AMF and a soil control produced from sterilized (120°C, 20 min) AMF mix inocula and a mix of the three local soils, respectively. Two hundred spores of each AMF species were used for the single AMF treatments, while a total of 100 spores of each AMF species were used to prepare the AMF mix. Due to high spore densities of *Cl. claroideum*, inocula were diluted with sterilized AMF mix inocula to add same inocula amounts as for *R. irregulare* and *Ce. helvetica*. The local soil treatments were supplemented with 15 g of pooled subplot soil per pot as described above. Since different inocula sources were added to the pots, their respective nutrient inputs were calculated but no relevant amounts were provided by the AMF or local soil inocula (nutrient analysis of all substrates can be found in Supplementary Table S1). All inocula were added to the planting hole before inserting the seedling. Each pot received 5 mL of a microbial wash to correct for possible differences in microbial communities (Koide and Elliott, 1989). The wash was prepared by wet sieving 100 g of each inoculum mix (either AMF- or local soil inocula mix for the respective treatments) through a 32 μm sieve and a paper filter (pore size 5–7 μm, FS 14 1/2; Schleicher & Schuell BioScience GmbH), yielding a final volume of 1 L. Plants were watered with distilled water according to their needs. Initially, plants were fertilized once after experimental start with 5 mL of a modified Hoagland solution containing 50% of the original P content (Gamborg and Wetter, 1975). After week 10, pots were fertilized weekly with 8 mL of the same Hoagland solution. Plants were grown in climate chambers (A. Schleiss AG, Magden, Switzerland) under controlled conditions with a 14 h light regime of 30000 lux, a temperature of 21–23°C and a relative humidity of 60–80%. To improve plant performance, growing conditions were modified after 14 weeks after transplantation into 1 L pots as follows: 14 h light regime of 60000 lux, a temperature of 23–25°C and a relative humidity of 80%. The experiment was carried out in a fully randomized design including 11 replicates per treatment.

#### Trap Culture Experiment

For trap culture establishment, the same plantlets and experimental substrate were used as described above. As inocula, soil of the four subplots per system was used directly without pooling to initiate trap cultures (Supplementary Figure S1). Naranjilla seedlings were grown for 6 month in 1 L plastic pots filled with 640 g of the experimental substrate. For inoculation, 50 g local soil inoculum of each subplot was spread in the planting hole before inserting the seedling. Irrigation and fertilization was performed in the same manner as the growth response experiment. Trap cultures were grown in the greenhouse with temperatures between 20 and 30°C and a relative humidity of 40–80%. The experiment was carried out in a fully randomized design including three replicates for each of the four subplot soil sample leading to a total of 12 replicates per naranjilla site (Supplementary Figure S1).

### Harvest and Measurements

#### Growth Response Experiment

After 14 weeks the first five pots of each treatment were harvested by cutting the plant shoot just above the soil surface. Before drying the shoot at 55°C for 48 h, maximum leaf length (length of the biggest leaf), shoot height (height of the uppermost node) and shoot fresh weight were measured. Dried shoots were weighed for dry weight estimation and subsequently milled in a coffee mill (Siemens MC23200, Typ KM13, BSH Hausgeräte GmbH, München, Germany). Shoot P concentration was measured using the molybdate blue method (Murphy and Riley, 1962) on a Segmented Flow Analyzer (Skalar Analytical B.V., San++ Automated Wet Chemistry Analyzer, Breda, Netherlands) after incineration and acid extraction of the plant powder. Nitrogen concentration was measured at the University of Basel using a Thermo Flash 2000 elemental analyzer (Carlo Erba, Thermo Fisher Scientific) where samples were combusted and C and N quantified using a thermal conductivity detector. The root system was carefully removed from the substrate, washed, weighed, cut into small pieces and split in two parts. The main part was dried at 55°C for 48 h to determine root dry weight while 1 g was kept to determine AMF root length colonization (RLC).

The same harvesting procedures were applied at the second harvest 22 weeks after experimental start with the remaining plants (six replicates).

#### Trap Culture Experiment

Six months after trap culture establishment four root and soil samples were taken from the periphery of each pot using a soil borer. After pooling, roots were removed from the soil and carefully washed. Three subsamples of about 200 mg and one subsample of about 1 g of fresh roots were stored at -20°C for later DNA extraction and RLC assessments, respectively. Remaining soil was mixed and also stored at -20°C for later DNA extraction.

#### Determination of AMF Root Length Colonization (RLC) from Growth Response and Trap Culture Experiments

Clean frozen root samples were stained to identify and count AMF structures inside the root by a method modified from Phillips and Hayman (1970). Roots were bleached in 10% KOH (10% in water, w:v) overnight at room temperature, rinsed with deionized water, neutralized with 1% HCl (1% in water, w:v) for 30 min and transferred to a ink-vinegar solution [57 mL Black Parker Quink in 1000 mL household vinegar (5% acetic acid), Migros, Switzerland] and stained overnight. Finally, roots were rinsed with deionized water and kept in 50% glycerol for destaining. The percentage of root length colonized by AMF was estimated by a modified line intersection method (McGonigle et al., 1990) at 200× magnification using Leitz Laborlux S microscope (Ernst Leitz Wetzlar GmbH, Germany). One hundred line-intersections per root sample were scored for AMF structures.

#### DNA extraction and AMF Community Profiling from Trap Culture Experiment

DNA extraction was performed as described by Green et al. (1999). In short, 2 mL of CTAB (cetyltrimethyl ammonium bromide) extraction buffer [100 mM Tris, 1.4 M NaCl, 50 mM Na_2_ EDTA, 2% (w/v) CTAB, 1% (w/v) PVP (K25 Roth, 4606)] were added to 200 mg of frozen trap culture roots and homogenized with the Homex 6 homogenizer (Bioreba AG, Reinach, Switzerland) in Bioreba extraction bags (Universal U-Unit, Art. Nr. 480100, Bioreba, Reinach, CH). Total genomic DNA was extracted using DNAeasy Plant Mini Kit (Qiagen, Hilden, Germany), following manufacturer’s recommendation. DNA concentration was measured with Qubit^TM^ 3.0 Fluorometer (ThermoFisher Scientific).

Root fungal community profiling was performed as described recently by Schlaeppi et al. (2016) with the exception that we could employ 1300 bp sequence information to infer operational taxonomic units (OTUs). We sequenced one SMRT^®^ Cells on a Sequel instrument at the Functional Genomic Centre Zurich^1^ (Zurich, Switzerland). The raw sequencing data is stored at the European Nucleotide Archive database (accession no. PRJEB20759). **Supplementary Data S1** documents the bioinformatic analysis (barcode-to-sample assignments and the command line script). The sequencing run produced a total of 171,673 raw reads with at least five passes (filtering by accuracy was not selected). After quality filtering, demultiplexing and the exclusion of samples that did not reach at least 400 quality sequences, we delineated OTUs at a level of 97% sequence similarity using usearch v8.0.1517 (Edgar, 2013). Taxonomic identities were assigned to the OTU representative sequences using the UNITE database (Kõljalg et al., 2013) with BLAST in the QIIME environment (Caporaso et al., 2010). We did not filter for AMF sequences [original approach by Schlaeppi et al. (2016)], because the mixed soil inocula for the trap cultures also contain fungi other than AMF. The final OTU table consisted of 31 samples (Conv: *n* = 11; Org: *n* = 9; Perm: *n* = 11), together with a total of 105,321 quality sequences (range 459–8,442, median 3,512 sequences per sample).

#### Statistical Analyses

Root length colonization data of both experiments and plant data of the growth response experiment were analyzed using one-way ANOVA (with inoculation as the main factor with nine and three levels in the growth response and trap culture experiment, respectively) followed by Tukey’s honest difference test with a significance level of α = 0.05. Normality of residuals was tested using Shapiro–Wilk test. RLC data were arcsin-square root transformed to fit the assumption of normal distribution. Correlations were calculated using Pearson’s correlation. Analyses were performed using JMP software version 11 (SAS, Cary, NC, United States).

We provide the statistical data analysis for fungal community profiling in R (v.3.2.2; R Core Team, 2014) including input files (OTU and taxonomy tables, OTU representative sequences and r-markdown script) as **Supplementary Data S2**. The OTU table was rarefied to the lowest sampling depth for ordination and richness analysis or, for the expression of relative abundance in %, it was normalized by sampling depth. β-diversity was assessed with Principal Coordinate Analysis (PCoA) and Permutational Multivariate Analysis of Variance (PERMANOVA), both analyses utilizing Bray–Curtis dissimilarity with packages include vegan (Oksanen et al., 2015) and phyloseq (McMurdie and Holmes, 2013).

## Results

### Response of Naranjilla to Inoculation with AMF and Local Soil Inocula

While maximum leaf length did not differ significantly between treatments at both harvests, shoot height was significantly affected by inoculation and differed between the first (*F* = 3.16, *p* = 0.0078) and second (*F* = 3.68, *p* = 0.0026) harvest (**Table 2**). At the first harvest, soil control plants were significantly taller than plants supplemented with conventional and permaculture soil, *R. irregulare* and the AMF mix. In contrast plants supplemented with Conv and Perm soil grew taller as compared to *R. irregulare*-inoculated plants. Similarly, shoot and root dry weights did not differ significantly between treatments, except for shoot dry weight at the first harvest (*F* = 2.5, *p* = 0.0282). Soil control plants had significantly higher shoot dry weights than *Cl. claroideum*-inoculated plants.

**Table 2:** Maximum leaf length, shoot height, shoot and root dry weights (DW) of naranjilla plants inoculated with different species of arbuscular mycorrhizal fungi (AMF), local soil inoculants from differentially managed Ecuadorian naranjilla plantations or two control treatments and grown for 14 weeks (WE 14) and 22 weeks (WE 22) in a sterilized potting substrate.

	Leaf length (cm)		Shoot height (cm)		Shoot DW (g)		Root DW (g)	
Treatment	WE 14	WE 22	WE 14	WE 22	WE 14	WE 22	WE 14	WE 22
*Rhizoglomus irregulare*	8.0	16.3	6.7 b	14.3 b	1.3 ab	5.44	0.57	1.89
*Claroideoglomus claroideum*	8.8	15.8	7.3 ab	15.3 ab	1.19 b	5.69	0.63	1.92
*Cetraspora helvetica*	8.3	16.7	7.4 ab	16.3 ab	1.55 ab	5.74	0.78	2.09
AMF mix	8.7	15.1	6.5 b	15.2 ab	1.23 ab	5.08	0.51	1.59
AMF control	7.8	15.5	7.3 ab	14.7 ab	1.45 ab	5.49	0.70	1.62
Conventional soil	8.0	16.1	6.6 b	16.8 a	1.69 ab	5.72	0.69	1.88
Organic soil	8.8	16.1	7.3 ab	16.3 ab	1.61 ab	6.17	0.64	1.74
Permaculture soil	8.5	15.2	6.8 b	16.9 a	1.53 ab	5.70	0.64	1.83
Soil control	8.5	15.1	8.8 a	15.0 ab	1.83 a	6.20	0.61	2.05
F_ANOV A_	0.82	0.69	3.16	3.68	2.5	1.05	1.38	2.26
*p*-Value	ns	ns	0.0078	0.0026	0.0282	ns	ns	ns

*Ns, not significant; AMF mix, mix of the three AMF species R. irregulare, Cl. claroideum, and Ce. helvetica; AMF control, sterilized inocula of the three AMF species; soil control, sterilized soil from all three naranjilla plantations. Different letters in the same column indicate significant different means [ANOVA, Tukey’s honest significant difference (HSD) test, α = 0.05]. Data represent means (n = 5 at WE 14 and 6 at WE 22).*

**FIGURE 1:**
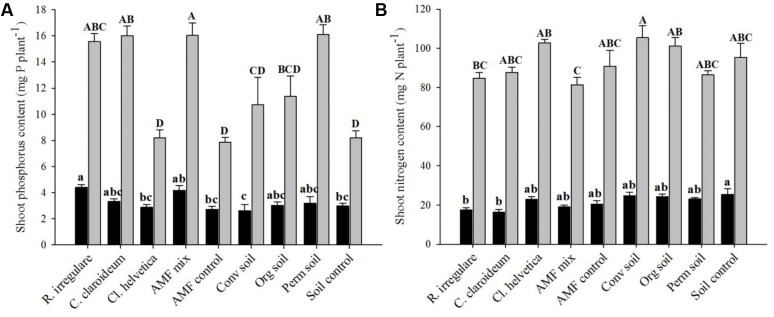
Phosphorus **(A)** and nitrogen **(B)** content of naranjilla shoots inoculated with different species of arbuscular mycorrhizal fungi (AMF): *Rhizoglomus irregulare*, *Claroideoglomus claroideum*, *Cetraspora helvetica*, a mix of the three AMF species and an AMF control consisting of a sterilized AMF inocula mix; or local soil inoculants derived from: conventional (Conv soil), organic (Org soil), and permaculture (Perm soil) naranjilla plantations in Ecuador and a sterilized mix of the three soils as soil control and grown for 14 weeks (WE 14) and 22 weeks (WE 22) in a sterilized potting substrate. Different lower and upper case letters above bars indicate significant differences at WE 14 and WE 22, respectively [ANOVA, Tukey’s honest significant difference (HSD) test, α = 0.05]. Data represent means + SE [*n* = 4 (for AMF control treatment due to exclusion of one contaminated sample), 5 (at WE 14), and 6 at (WE 22)].

Inoculation of naranjilla significantly affected shoot P content at the first and second harvest (*F* = 14.06 and *F* = 11.84, *p* = 0.023 and *p* < 0.0001, respectively). After 14 weeks plants inoculated with *R. irregulare* contained more P in shoots than plants inoculated with *Ce. helvetica*, Conv soil and the two control treatments. After 22 weeks, differences between treatments increased and all reference AMF treatments (except of *Ce. helvetica*) and Perm soil treatment revealed a significantly higher P content than the control treatments (**Figure 1A**). Inoculation with *R. irregulare* and the AMF mix significantly increased shoot P concentrations (*F* = 14.6, *p* < 0.0001) at the first harvest while inoculation with *Cl. claroideum*, *R. irregulare*, the AMF mix, and Perm soil significantly increased shoot P concentration (*F* = 15.4, *p* < 0.0001) at the second harvest (Supplementary Table S2).

Also shoot N content was significantly affected by inoculation at the first and second harvest (*F* = 3.81 and *F* = 4.31, *p* = 0.024 and *p* = 0.0009, respectively). Highest shoot N contents were measured in soil control plants at the first harvest and plants supplemented with Conv soil at second harvest (**Figure 1B**). However, no significant differences were found between AMF treatments and control treatments. Shoot N concentrations were similar in all treatments at both harvests (Supplementary Table S2).

**FIGURE 2:**
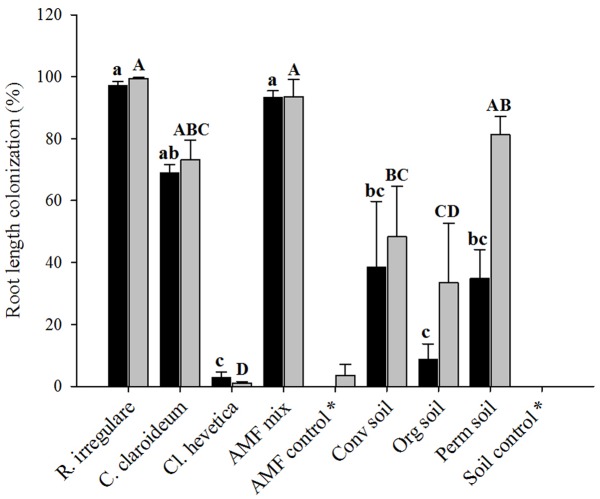
Root length colonization of naranjilla roots after 14 (black bars) and 22 (gray bars) weeks of growth as affected by inoculation with different species of AMF: *Rhizoglomus irregulare*, *Claroideoglomus claroideum*, *Cetraspora helvetica*, a mix of the three AMF species and an AMF control consisting of a sterilized AMF inocula mix; or local soil inoculants derived from: conventional (Conv soil), organic (Org soil), and permaculture (Perm soil) naranjilla plantations in Ecuador and a sterilized mix of the three soils as soil control. Different lower and upper case letters above bars indicate significant differences at WE 14 and WE 22, respectively (ANOVA, Tukey’s HSD test, α = 0.05). Treatments with an asterisk were excluded from statistical analysis. Data represent means + SE [*n* = 4 (for AMF control treatment due to exclusion of one contaminated sample), 5 (at WE 14), and 6 at (WE 22)].

Root length colonization by AMF significantly correlated with shoot P content after 14 weeks (*r* = 0.6783, *p* > 0.001) and 22 weeks (*r* = 0.8475, *p* < 0.0001) and was significantly affected by inoculation at both harvests (*p* < 0.0001). Plants inoculated with *R. irregulare* and the AMF mix showed the highest RLC rates while roots of *Ce. helvetica*-inoculated plants remained weakly colonized at both harvest time points (**Figure 2**). For most of the AMF treatments, RLC rates did not change between both harvests, only for the two local soil inocula, Org and Perm soil, RLC rates highly increased from the first to the second harvest. Roots of the control treatments remained in most cases free of mycorrhizal structures. Nevertheless one plant of the soil control (first harvest) and one plant of the AMF control treatment (second harvest) had roots highly colonized, most likely due to cross-contaminations and were consequently excluded from graphical representation and all statistical analyses. Roots of another AMF control plant contained at the second harvest only few hyphae but no arbuscules nor vesicles and hence this replicate was kept for further analyses.

### AMF Communities in Differentially Managed Naranjilla Plantations

We conducted a trap culture experiment to characterize the AMF communities in the differentially managed soils from the naranjilla plantation site in Guamaní. After 6 month of growth in the greenhouse, RLC of trap cultured naranjilla plants significantly differed between the three local soil inocula (*F* = 10.3, *p* = 0.0003) and was the highest for plants inoculated with Perm soil (44%) compared to plants inoculated with Org soil (21%) and Conv soil (9%).

**FIGURE 3:**
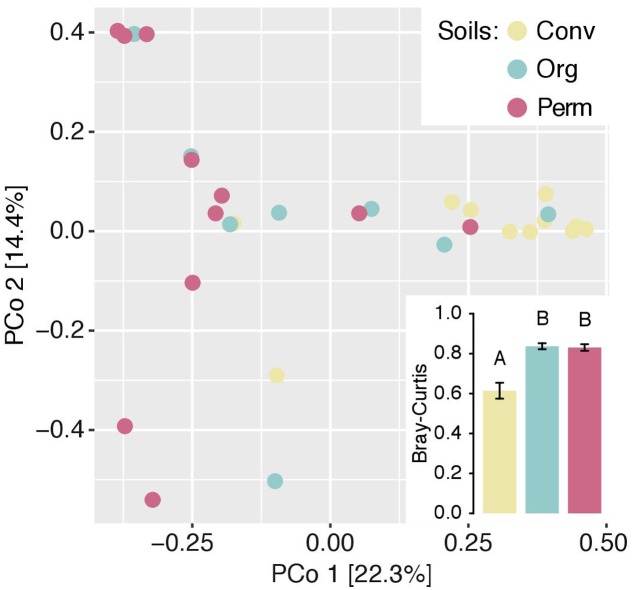
Variation between naranjilla root communities from trap cultures. Differences between communities were assessed with principal coordinate analysis (PCoA) based on Bray–Curtis dissimilarity. Points in the unconstrained ordination represent individual samples and are colored by the type of soil used for the trap cultures. Data was subsampled to the sequence number of the sample with the lowest sampling depth. Percentage of variation given on each axis refers to the explained fraction of total variation in all samples. Inset: Average dissimilarity within groups. Each sample was compared with all other samples within its group and these Bray–Curtis dissimilarity were averaged for each sample and plotted by soil groups.

We determined the fungal taxa inside the roots of the trap culture plants based on amplicon sequencing. Initially, we compared the between-sample similarities based on the β-diversity index Bray–Curtis. PERMANOVA revealed that the fungal communities differed significantly between the different soil types (*F*model = 2.484, *p* = 0.002; Supplementary Table S3). Unconstrained ordination supports this finding with separating the samples of the three different soil groups along Axis 1 (**Figure 3**, explaining 22.3% of the variation). We noted that the root fungal communities trapped from organic and permaculture soils were significantly more heterogeneous compared to those trapped from conventional soil (**Figure 3**, inset).

**FIGURE 4:**
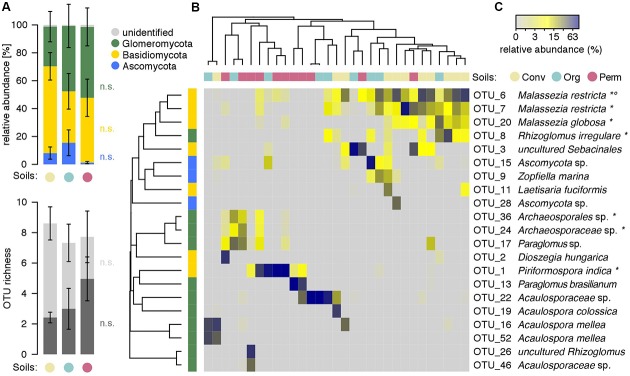
Root fungi profiles from trap cultures. **(A)** Mean abundances of fungal phyla plotted by soil groups. Data were normalized by sampling depth and expressed as percentage relative abundance. **(B)** Mean fungal (light gray) and Glomeromycota (dark gray) OTU richness, reported by soil groups. The dataset was independently subsampled 100 times, OTU richness determined and averaged for each sample. **(C)** The heatmap displays all abundant (defined as >1% mean relative abundance) OTUs in percentage relative abundance in all samples. Dendrograms summarize similarities between samples (in columns) and between OTUs (in rows) based on Bray–Curtis. Non-parametric Kruskal–Wallis test was used to examine differences between soil groups (n.s., not significant, ^∗^*P* < 0.05, °*P* < 0.05 false discovery rate corrected).

Closer inspection of the root fungi profiles revealed that the communities trapped from the differently managed soils differed in their composition (**Figure 4**). Of note, the high variation between replicate samples, especially the ones derived from organic and permaculture soils, precluded statistical support for these differences. Basidiomycetes and Ascomycetes were more abundant in root fungal communities trapped from conventional and organic soils, respectively. Consistent with the AMF RLC analysis, we found increasing abundances of AMF in roots if trapped from conventional, organic to permaculture soils (**Figure 4A**). This observation was also reflected in the number of detected AMF sequence groups (aka OTUs, operational taxonomic units): while the communities did not differ in the overall number of detected OTUs, we noted increasing numbers of AMF OTUs in roots if trapped from conventional, organic to permaculture soils (**Figure 4B**).

We defined ‘abundant OTUs’ when OTUs were reaching a minimal mean abundance of 1% in the dataset and we examined their distribution pattern in the root fungi profiles from the trap cultures (**Figure 4C** and Supplementary Figure S2). While numerous communities from conventional or permaculture soils tended to cluster with their group (indicative for similar community composition), we did not find a clear pattern for the root fungi profiles from the trap cultures with organic soil. We found mainly in roots of organic soil trap cultures abundant *Malassezia* spp. (Phylum Basidiomycota) and this, often in combination with the AMF fungus *R. irregulare*. In contrast, we noted predominantly in root samples of permaculture soil diverse AMF taxa such as *Acaulospora* or *Paraglomus* spp.. Interestingly, the beneficial Basidiomycete *Piriformospora indica* was primarily abundant and almost exclusively found in roots of Permaculture soil trap cultures.

## Discussion

The application of AMF in horticulture became more prominent within the last decades as the number of studies demonstrating improved plant growth after inoculation has steadily increased (Requena et al., 2001; Gianinazzi et al., 2012). The application of AMF in nurseries which involves growing plants in the presence of AMF prior to planting them in the field is especially beneficial since (i) growing substrates regularly lack natural soil microorganisms, (ii) the plants possess an already established mycorrhizal symbiosis for improving the plants’ tolerance against biotic and abiotic stresses when transplanted to the field, and (iii) hyphal colonization of the soil and hyphal nutrient assimilation can start immediately after field transplantation. Thus, the application of AMF harbors a huge potential especially during the early stages of crop cultivation. At later stages, soil fertility becomes crucial for the crops’ performance and many studies report that this fertility is more often associated with less intensive practices involving abundant and diverse microbial communities (Van der Heijden et al., 2008; Wagg et al., 2014). In the present study we aimed at investigating both aspects, the potential of AMF to improve plant growth and nutrition and the impact of different farming practices on soil fungal communities, in relation to the cultivation of naranjilla, an important cash crop in Ecuador, Colombia, and Central America.

### Response of Naranjilla to Inoculation with AMF and Local Soil Inocula

We investigated the impact of inoculation of naranjilla with reference AMF strains and compared this with a novel inoculation method using natural soils originating from three differentially managed agricultural systems in Ecuador. Growth parameters, biomass production, and shoot nutrient content were measured twice, after 14 and 22 weeks, and revealed that successfully colonized plants were able to acquire more P in their shoots. The strong positive correlation of shoot P content and mycorrhizal RLC raised evidence for naranjillas’ dependency on the association with AMF for nutrient acquisition which confirms findings of previous studies. Gonzalez and Osorio (2015) observed that *Glomus aggregatum* increased P uptake and biomass production of inoculated plants compared to non-mycorrhizal controls when grown under greenhouse conditions. Also, the commercial inoculum Mycobiol^®^, consisting of *Glomus* spp., *Entrophospora colombiana*, and *Acaulospora mellea* enhanced P acquisition and plant growth in a pot experiment (Casierra-Posada et al., 2013). In the present study inoculation with the reference AMF species *R. irregulare*, *Cl. claroideum*, and the AMF mix improved P uptake by the plants. Though not only pure AMF inocula also inoculation with natural soil from a naranjilla permaculture system enhanced P uptake to a similar degree. The approach of using local soils as inoculants has not yet been used for crop plant cultivation before. Up to date experience has been gained using this type of inoculant with few examples related to the cultivation of tropical tree seedling. In a study performed by Urgiles et al. (2014) inoculation with local forest soil was tested for its efficiency to promote the growth of two pioneer trees native to the tropical mountain rain forest in the Andes of Ecuador. The study revealed that application of local forest soil increased seedlings growth to a greater extent than application of a pot-produced inoculant consisting of native AMF species. The authors suggested that beside native AMF populations present in the soil potentially also other beneficial soil microorganisms accounted for improved seedlings performance. Evidence for a similar effect has been made in our study. Also, Rowe et al. (2007) observed improved performance of late successional tree seedlings after inoculation with field soil as compared to inoculation with two different commercial inoculants. Beside enhanced seedlings growth also the colonization success in terms of RLC was significantly higher when field soil was used.

In accordance to the above mentioned studies we could also demonstrate that the application of local soils as inoculant represents a valid alternative to the use of commercial AMF products especially for ecosystems harboring healthy and microbial active soils. The approach of using local soils offers multiple advantages compared to the use of commercial inoculants. Firstly, the application of local soils helps to conserves the local below-ground biodiversity. Nowadays, the use of commercial AMF inoculants is growing and certain AMF species (mainly *R. irregulare*) are traded globally and used in agriculture and revegetation programs (Gianinazzi et al., 2012) even if they are not native in the applied soils. How introduced AMF species would alter existing AMF communities is still poorly understood as only few greenhouse studies have addressed this question. While some studies detected a decrease in diversity (Mummey et al., 2009; Koch et al., 2011) or functionality (Symanczik et al., 2015) of native AMF communities by introduced AMF inoculants, other studies observed no effect (Antunes et al., 2009). Without more precise knowledge about potential changes of native communities due to current application practices, there remains a risk of future unwanted consequences (Schwartz et al., 2006). Secondly it has been suggested that native AMF strains are better adapted to the local environmental conditions and thus better promote plant growth. A meta-study analyzing the influence of inoculum source revealed that inoculants consisting of local AMF strains yielded higher increases in mycorrhizal colonization and plant growth responses than inoculants from commercial sources (Maltz and Treseder, 2015). Moreover, the use of local soils from reference ecosystems as inocula is economically advantageous especially for smallholder farmers who otherwise might not be able to purchase expensive commercial inocula. Furthermore, the availability of high quality inoculants has to be considered in this context. A study of Corkidi et al. (2004) investigated the infectivity of 10 commercially available mycorrhizal inoculants by inoculating maize plants with recommended rates of each inoculant type. The observed percentages of root colonization ranged from highly insufficient (0%) to satisfactory (50%) and was explained by the presence or absence of viable propagules, the content and type of infective propagules, the amount of recommended application rates as well as unbeneficial soil-microbial interactions. This study indicates possible restrictions which might be associated with the use of commercial mycorrhizal inoculants.

Results from the growth response experiment highlight the potential of using local soils as inoculants especially when other sources of native AMF strains are unavailable. However, we also need to point out the importance of testing a soil before its application to guarantee the absence of pathogens and other growth inhibiting substances. Additional experiments using this source of inoculant are needed to verify our observations and to make more general predictions about the potential.

### Root Fungal Communities Trapped from Differentially Managed Naranjilla Plantations

In a second step, we analyzed the root fungal communities trapped from the three differentially managed soils with a focus on AMF. Root analysis revealed that fungal communities significantly differ among the sites with Org and Perm soils harboring more heterogeneous communities than Conv soil. Since separate and non-homogenized soil samples were used as input for the trap cultures, we probably observe in the roots the local soil heterogeneities from the plots from which soil samples had been collected. These observations likely indicate that Org and Perm plots were more heterogeneous and thus more diverse than Conv plots. The higher heterogeneity of the fungal communities might be explained by the higher above ground diversity within these two types of management systems. The influence of plant community composition on belowground microorganisms has already been shown in the case of AMF. Several studies found a positive correlation between plant diversity and AMFs’ species richness and abundance (Johnson et al., 2004; Lovelock and Ewel, 2005; Krüger et al., 2017). Similarly we have observed that AMF OTUs and mycorrhizal RLC steadily increased in roots when trap cultures were supplemented with soils from conventional to organic to those from permaculture systems. Also Oehl et al. (2003) observed that management systems with a high plant species richness were correlated with an increase in AMF species richness and abundance. Compared were three management systems: low-input, species-rich grassland; low- to moderate-input farming with a 7-year crop rotation and high-input, continuous maize mono-cropping. Results have shown that some AMF almost exclusively species from the genus of *Glomeraceae* occurred within all management systems, but that the majority of AMF was either restricted to grasslands or less intensively farmed systems with crop rotation especially species of the genera *Acaulosporaceae* and *Scutellosporaceae*. Similarly we have detected that *Acaulospora* spp. were almost exclusively present in roots supplemented with Perm soil, the management system with the highest above ground biodiversity.

It is well established that agricultural management practices highly impact AMF but also other fungal communities and that less intense farming systems favor a higher functionality, abundance, and diversity (Mäder et al., 2000; Oehl et al., 2003, 2004; Gosling et al., 2006; Verbruggen et al., 2010; Kalia and Gosal, 2011; Köhl et al., 2014). One important type of soil disturbance which has been shown to strongly affect soil fungal communities is the application of pesticides as reviewed by Kalia and Gosal (2011). Fungicides directly impact soil fungi with AMF being the most vulnerable candidates. Several studies have reported about fungicides’ negative impacts like a decrease in hyphal growth and sporulation of AMF (Sukarno et al., 1993; Kurle and Pfleger, 1994; Chiocchio et al., 2010) a reduction in mycorrhizal root colonization rates (Abd-Alla et al., 2000) as well a decrease in overall fungal biomass of 85% (Bossuyt et al., 2001). Similarly extensive applications of P fertilizers has been shown to affect mycorrhizal performance in a similar manner as reviewed by Jansa et al. (2006). The conventional naranjilla plantation from which the soil of this study was collected, regularly received high levels of external inputs like synthetic fertilizers and a cocktail of different pesticides being most likely the reason for the observed loss in heterogeneity and reduction of AMF abundance and diversity.

Our NGS approach revealed that *Piriformospora indica* was only detected in roots of trap cultures supplemented with permaculture soil. A review by Varma et al. (2012) highlighted the multifunctional role of *P. indica* as plant growth promoting fungus. Besides its abilities to promote plant growth (Achatz et al., 2010; Fakhro et al., 2010) and nutrient provisioning (Yadav et al., 2010), *P. indica* was also shown to confer tolerance against biotic and abiotic stresses (Fakhro et al., 2010; Sun et al., 2010; Kumar et al., 2012). Improved P acquisition by naranjilla in the growth response experiment might also have resulted from the interaction of this fungus with naranjilla and/or native AMF strains of the Perm soil.

Further, we identified abundant sequences that map taxonomically to *Malassezia* spp., i.e., *M. globosa* and *M. restricta* mainly inhabiting naranjilla roots supplemented with the Conv soil. A review by Amend (2014) described the two *Malassezia* spp. which were originally known as mammalian skin pathogens as cosmopolitan organisms present in different marine habitats, on the exoskeleton of nematodes and in Antarctic soils. Only one study performed by Roy et al. (2009) reported the occurrence of *Malassezia* spp. colonizing the roots of orchids sampled in the rainforest of Thailand. However, the relevance of this association with plant roots and potential impacts on plant growth performance is not known and would require further research including isolation and characterization of strains of this fungal group.

The high homogeneity in soil fungal communities and the loss in AMF abundance in the conventional system compared to the organic one once more demonstrates how strong and rapid intensive management practices can affect soil biota, considering that only one and 1.5 year passed after converting the natural forest into the conventional and organic plantations, respectively. In this way, our findings highlight again the importance of highly diverse belowground systems including AMF for the stability and productivity of agroecosystems and ecosystems’ multifunctionality (Van der Heijden et al., 2008; Wagg et al., 2014). Indeed, belowground systems characterized by a low biodiversity are more susceptible to the spread of diseases (Brussaard et al., 2007) and nutrient leaching (Wagg et al., 2014) and harbor a lower potential for nutrient cycling (De Vries et al., 2013; Wagg et al., 2014). Hence, less intensive cultivation systems like permacultures and agroforestry as well as organic farming should be favored to promote divers below-ground soil-microbial communities.

## Conclusion

Our study is the first of its kind comparing the effectiveness of exotic AMF strains versus local soil inoculation for naranjilla cultivation with a combined assessment of soil fungal communities in differentially managed naranjilla plantations. This study shows the potential of combining both the use of local soils as inoculants at the nursery stage and the application of less intensive practices as a promising approach to improve and maintain sustainable and resilient naranjilla production systems.

## Author Contributions

SS was the leading author and contributed to all study related tasks mentioned below. CT contributed to research and supervised MG. The M.Sc. student MG performed all practical work in the frame of her M.Sc. thesis. AK, KS, and MV contributed in research and editing of the manuscript. PM and TB are senior author who guided the research and contributed by editing of the manuscript.

## Conflict of Interest Statement

The authors declare that the research was conducted in the absence of any commercial or financial relationships that could be construed as a potential conflict of interest.

## Footnotes

1 www.fgcz.ch

## Supplementary Material

DATA S1 Bioinformatic analysis files.

DATA S2 Statistical data analysis files for fungal community profiling in R.
