# Supplementary material for: Application of Mycorrhiza and Soil from a Permaculture System Improved Phosphorus Acquisition in Naranjilla
Source: Front Plant Sci. 2017 Jul 19;8:1263. doi: 10.3389/fpls.2017.01263 (PMC5515901; doi:10.3389/fpls.2017.01263)
Supplement: DATA S2 — Statistical data analysis files for fungal community profiling in R. [file Data_Sheet_3.ZIP › Supplementary_Data_S2/Symanczik_et_al.pdf]

Symanczik et al. (2017), R-Analysis

Klaus Schlaeppi

2017-05-03

Contents

data import **2**
sequence numbers . . . 2
sequence numbers by sample groups . . . 3

taxonomy **3**

beta diversity analysis **4**
PCoA . . . 4
bray curtis within groups . . . 5
PERMANOVA . . . 6

PHYLUM level analysis **7**
matrix at phylum level . . . 7
Ascomycota statistics . . . 7
Basidiomycota statistics . . . 7
Glomeromycota statistics . . . 8

OTU level analysis **11**
Defining abundant OTUs (>1%) . . . 11
OTUs of differential abundance . . . 11
Displaying abundant OTUs . . . 12
OTU richness . . . 13
Glomeromycota OTU richness . . . 14

data import

sequence numbers

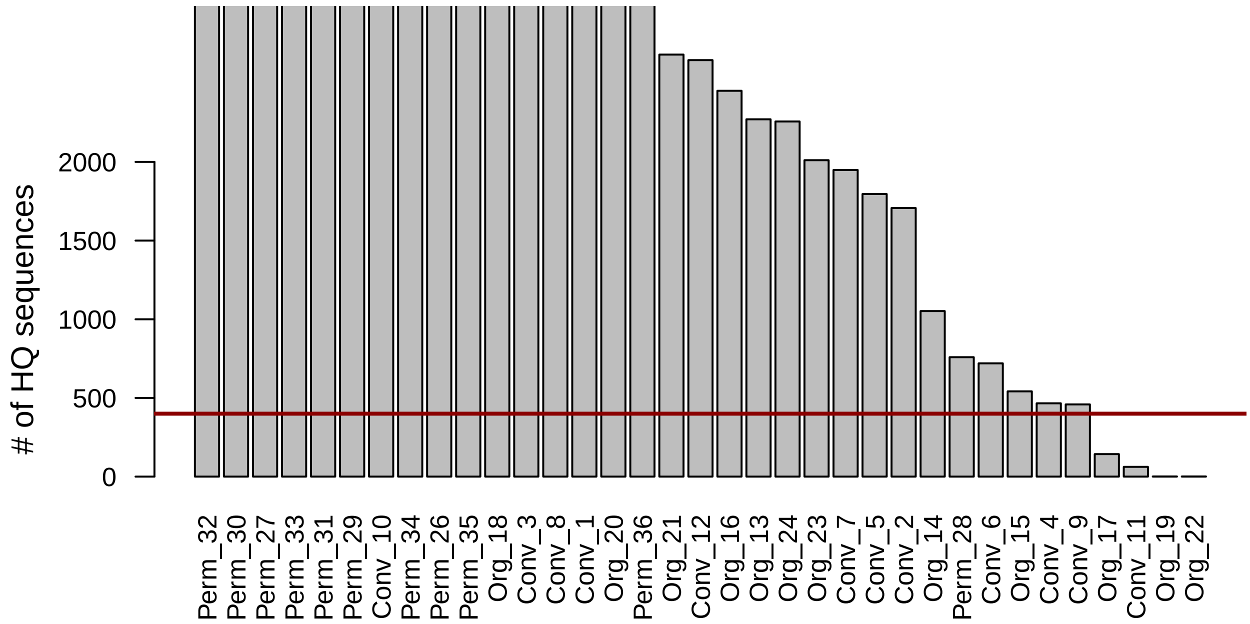


```
## [1] 105321
```

```
## [1]  459 8442
```

```
## [1] 3512
```

We subsetted the otu table to samples that had at least 400 sequences.

sequence numbers by sample groups

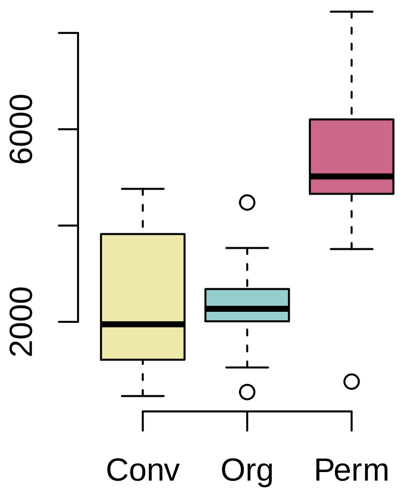


```
##
##  Kruskal-Wallis rank sum test
##
## data:  colSums(dat) by design[colnames(dat), ]$Soil
## Kruskal-Wallis chi-squared = 12.478, df = 2, p-value = 0.001952
```

significant differences between sequence numbers per sample groups. Therefore, use rarefication for normalization of the data (see Weiss et al., 2015 PeerJ)

taxonomy

```
##
##         Fungi No blast hit      Plantae     Protista
##            74            2            1            6
```

We subsetted the otu table to fungi OTUs only.

beta diversity analysis

PCoA

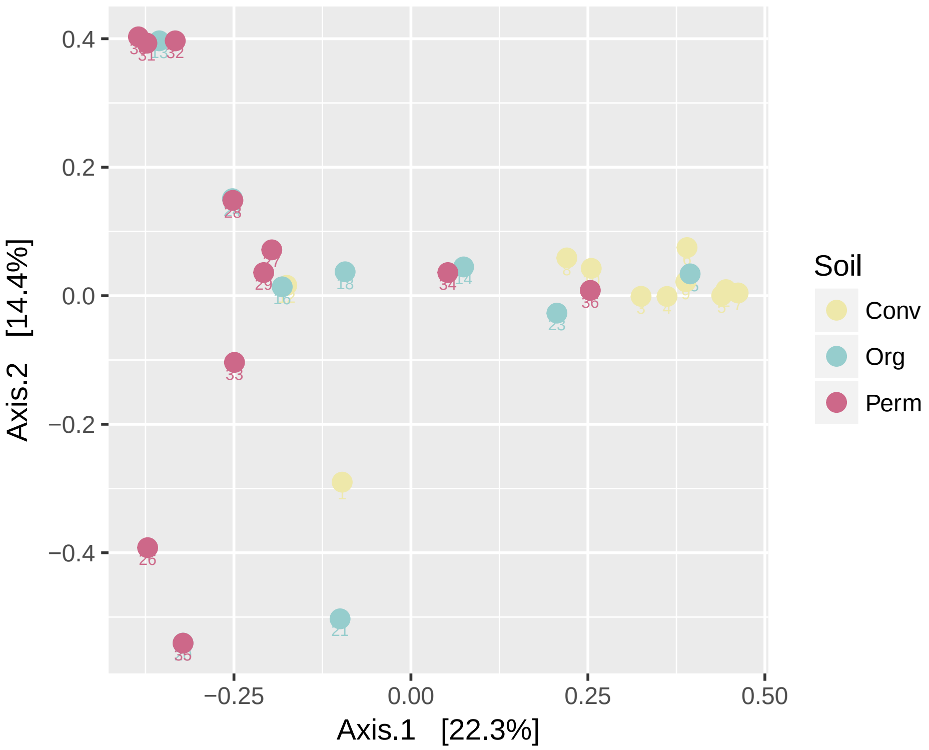


```
## pdf
##   2
```

bray curtis within groups

```
##   Kruskal-Wallis rank sum test
##
## data: x and group
## Kruskal-Wallis chi-squared = 13.2283, df = 2, p-value = 0
##
##
##                            Comparison of x by group
##                              (Benjamini-Hochberg)
## Col Mean-|
## Row Mean |       Conv        Org
## ---------+----------------------
##      Org |  -3.126746
##          |    0.0027*
##          |
##     Perm |  -3.118710   0.168078
##          |    0.0014*     0.4333
```

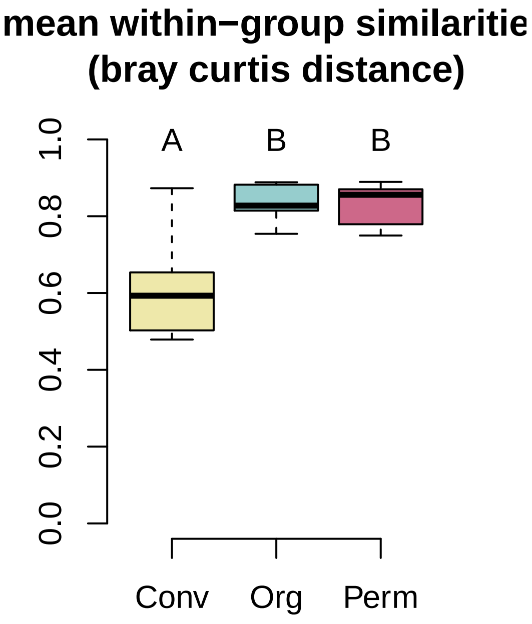

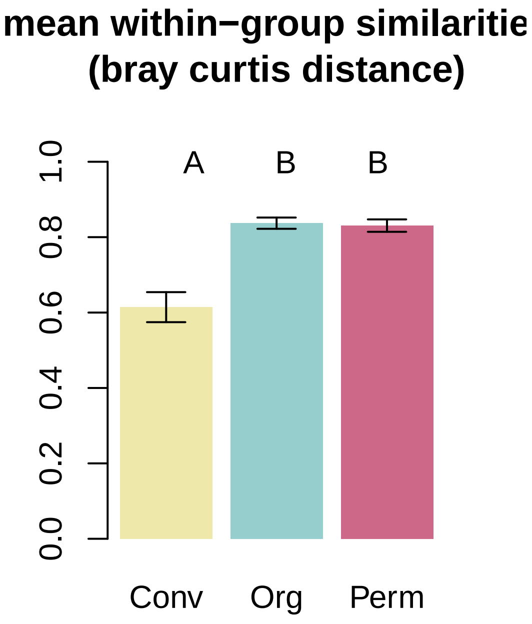


```
## pdf
##   2
```

PERMANOVA

	Df	SumsOfSqs	MeanSqs	F.Model	R2	Pr(>F)
**Soil**	2	1.866	0.9331	2.484	0.1507	0.002
**Residuals**	28	10.52	0.3756	NA	0.8493	NA
**Total**	30	12.38	NA	NA	1	NA

PHYLUM level analysis

matrix at phylum level

```
##
## Glomeromycota    Ascomycota Basidiomycota  unidentified
##            28            17            17            12

##               cAMF18F2R1 cAMF18F3R1 cAMF18F5R1 cAMF18F6R1 cAMF18F7R1
## Ascomycota            34          0         99          2         31
## Basidiomycota       1233       4088       1443        555       1089
## Glomeromycota         69          1        112        122         84
## unidentified           3          1         20         11         14
```

Ascomycota statistics

```
##      Conv       Org      Perm
##  8.093045 15.487186  1.264928

##
##  Kruskal-Wallis rank sum test
##
## data:  PHYLUM_mat_norm["Ascomycota", ] by Soil
## Kruskal-Wallis chi-squared = 4.0856, df = 2, p-value = 0.1297

##   Kruskal-Wallis rank sum test
##
## data: x and group
## Kruskal-Wallis chi-squared = 4.0856, df = 2, p-value = 0.13
##
##
##                           Comparison of x by group
##                             (Benjamini-Hochberg)
## Col Mean-|
## Row Mean |       Conv        Org
## ---------+----------------------
##      Org |  -0.083513
##          |     0.4667
##          |
##     Perm |   1.738280   1.732590
##          |     0.1232     0.0624
```

Basidiomycota statistics

```
##     Conv      Org     Perm
## 62.20439 36.89068 46.47108

##
##  Kruskal-Wallis rank sum test
##
## data:  PHYLUM_mat_norm["Basidiomycota", ] by Soil
## Kruskal-Wallis chi-squared = 1.9945, df = 2, p-value = 0.3689
```

```
##   Kruskal-Wallis rank sum test
##
## data: x and group
## Kruskal-Wallis chi-squared = 1.9945, df = 2, p-value = 0.37
##
##
##                            Comparison of x by group
##                              (Benjamini-Hochberg)
## Col Mean-|
## Row Mean |       Conv        Org
## ---------+----------------------
##      Org |   1.397094
##          |     0.2436
##          |
##     Perm |   0.844502  -0.595928
##          |     0.2988     0.2756
```

Glomeromycota statistics

```
##     Conv      Org     Perm
## 28.46565 46.90399 50.83767
```

```
##
##  Kruskal-Wallis rank sum test
##
## data:  PHYLUM_mat_norm["Glomeromycota", ] by Soil
## Kruskal-Wallis chi-squared = 1.2549, df = 2, p-value = 0.5339
```

```
##   Kruskal-Wallis rank sum test
##
## data: x and group
## Kruskal-Wallis chi-squared = 1.2549, df = 2, p-value = 0.53
##
##
##                            Comparison of x by group
##                              (Benjamini-Hochberg)
## Col Mean-|
## Row Mean |       Conv        Org
## ---------+----------------------
##      Org |  -0.872875
##          |     0.2870
##          |
##     Perm |  -1.032170  -0.106327
##          |     0.4530     0.4577
```

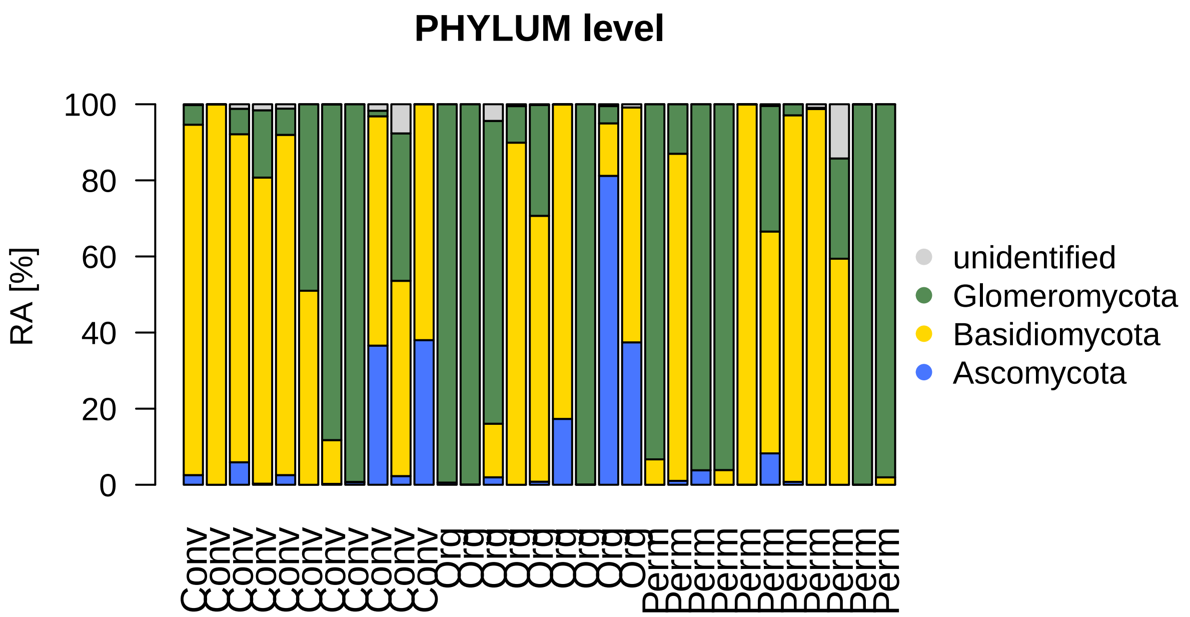
PHYLUM level
RA [%]
0
20
40
60
80
100
unidentified
Glomeromycota
Basidiomycota
Ascomycota

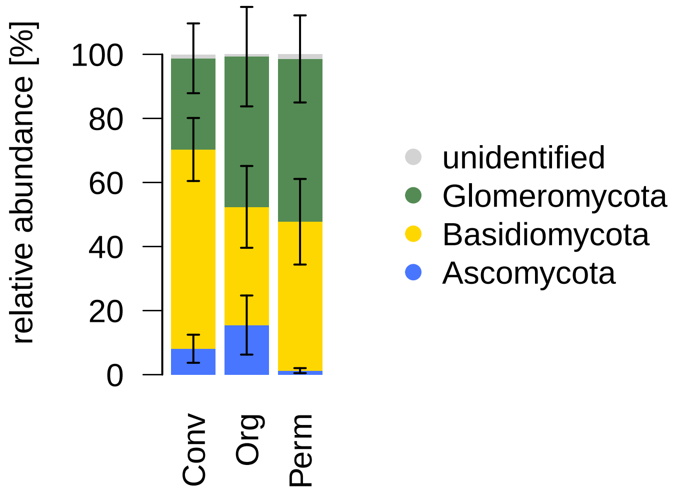
relative abundance [%]
0
20
40
60
80
100
Conv
Org
Perm
unidentified
Glomeromycota
Basidiomycota
Ascomycota

```
## pdf
##   2
```

Glomeromycota

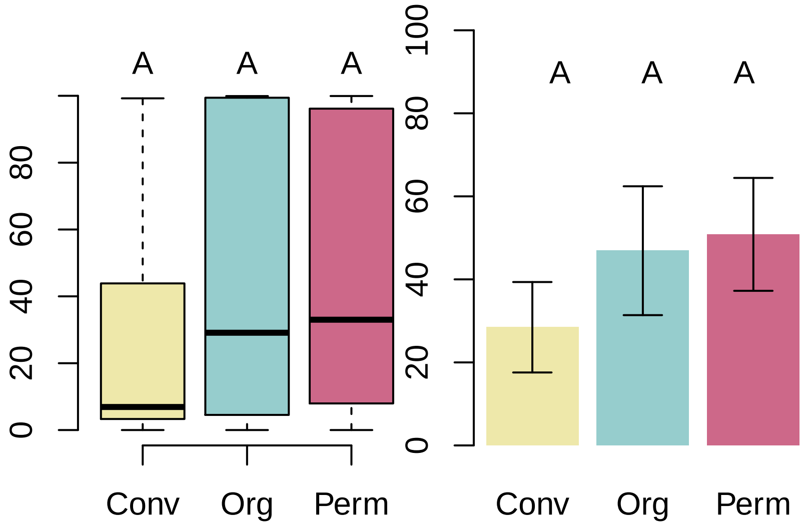

OTU level analysis

Defining abundant OTUs (>1%)

OTUs of differential abundance

```
## pdf
##   2
```

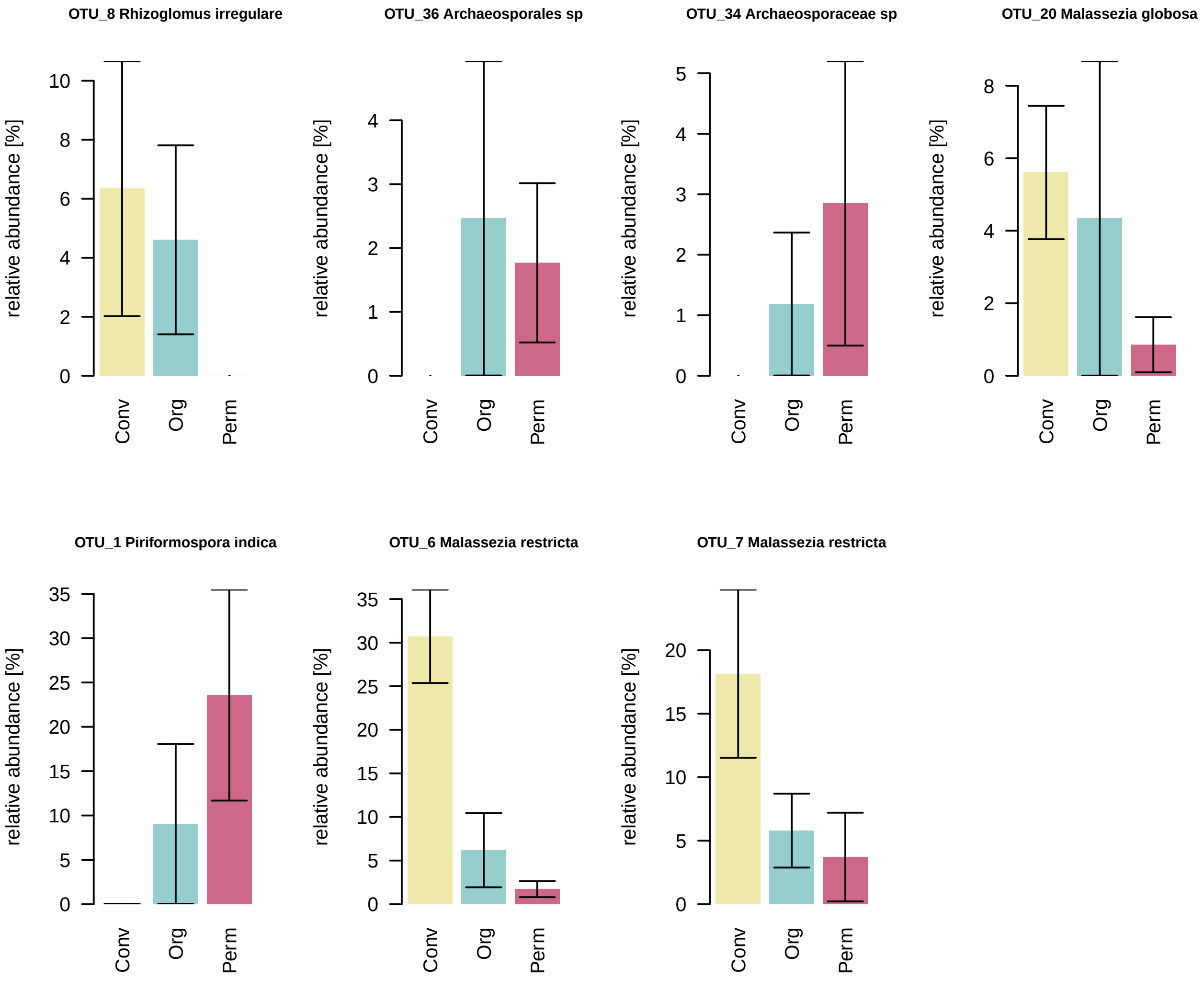

Displaying abundant OTUs

```
##
## Attaching package: 'gplots'

## Das folgende Objekt ist maskiert 'package:stats':
##
##     lowess
```

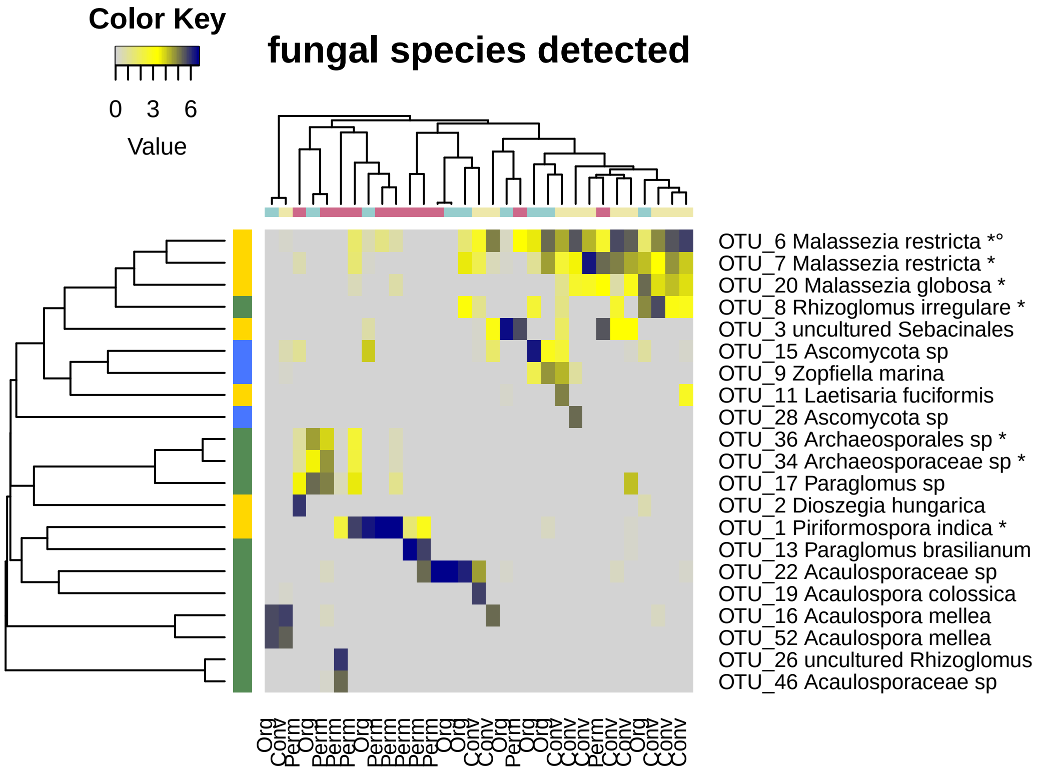


```
## pdf
##   2
```

OTU richness

```
##   Kruskal-Wallis rank sum test
##
## data: x and group
## Kruskal-Wallis chi-squared = 1.0171, df = 2, p-value = 0.6
##
##
##                             Comparison of x by group
##                               (Benjamini-Hochberg)
## Col Mean-|
## Row Mean |       Conv        Org
## ---------+----------------------
##      Org |   0.724218
##          |     0.3517
##          |
##     Perm |   0.961407   0.187851
##          |     0.5045     0.4255
```

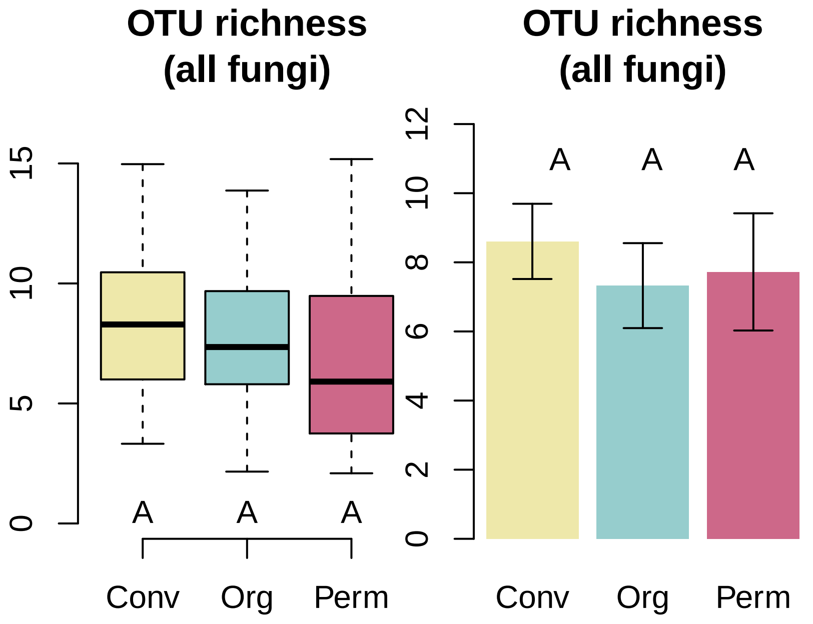

Glomeromycota OTU richness

```
##   Kruskal-Wallis rank sum test
##
## data: x and group
## Kruskal-Wallis chi-squared = 1.2822, df = 2, p-value = 0.53
##
##
##                            Comparison of x by group
##                              (Benjamini-Hochberg)
## Col Mean-|
## Row Mean |       Conv         Org
## ---------+----------------------
##      Org |   0.744629
##          |     0.3424
##          |
##     Perm |  -0.305887  -1.121588
##          |     0.3798     0.3931
```

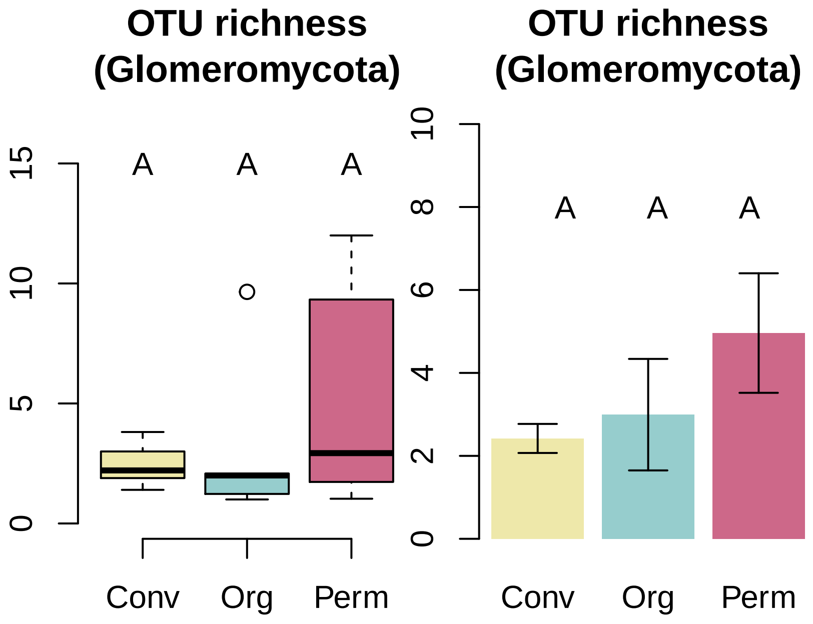


```
## pdf
##   2
```
