# Supplementary material for: Application of Mycorrhiza and Soil from a Permaculture System Improved Phosphorus Acquisition in Naranjilla
Source: Front Plant Sci. 2017 Jul 19;8:1263. doi: 10.3389/fpls.2017.01263 (PMC5515901; doi:10.3389/fpls.2017.01263)
Supplement: Supplementary file 3 [file Data_Sheet_1.DOCX]

Supplementary Material

Application of mycorrhiza and soil from a permaculture system improved phosphorus acquisition in naranjilla

**Sarah Symanczik^*^, Michelle Gisler, Cécile Thonar, Klaus Schlaeppi, Marcel van der Heijden, Ansgar Kahmen, Thomas Boller, Paul Mäder**

*** Correspondence:** Sarah Symanczik: [sarah.symanczik@fibl.org](mailto:sarah.symanczik@fibl.org)

# Supplementary Data

Supplementary Data S1 and S2 were uploaded separately as zip files.

# Supplementary Figures and Tables

## Supplementary Figures


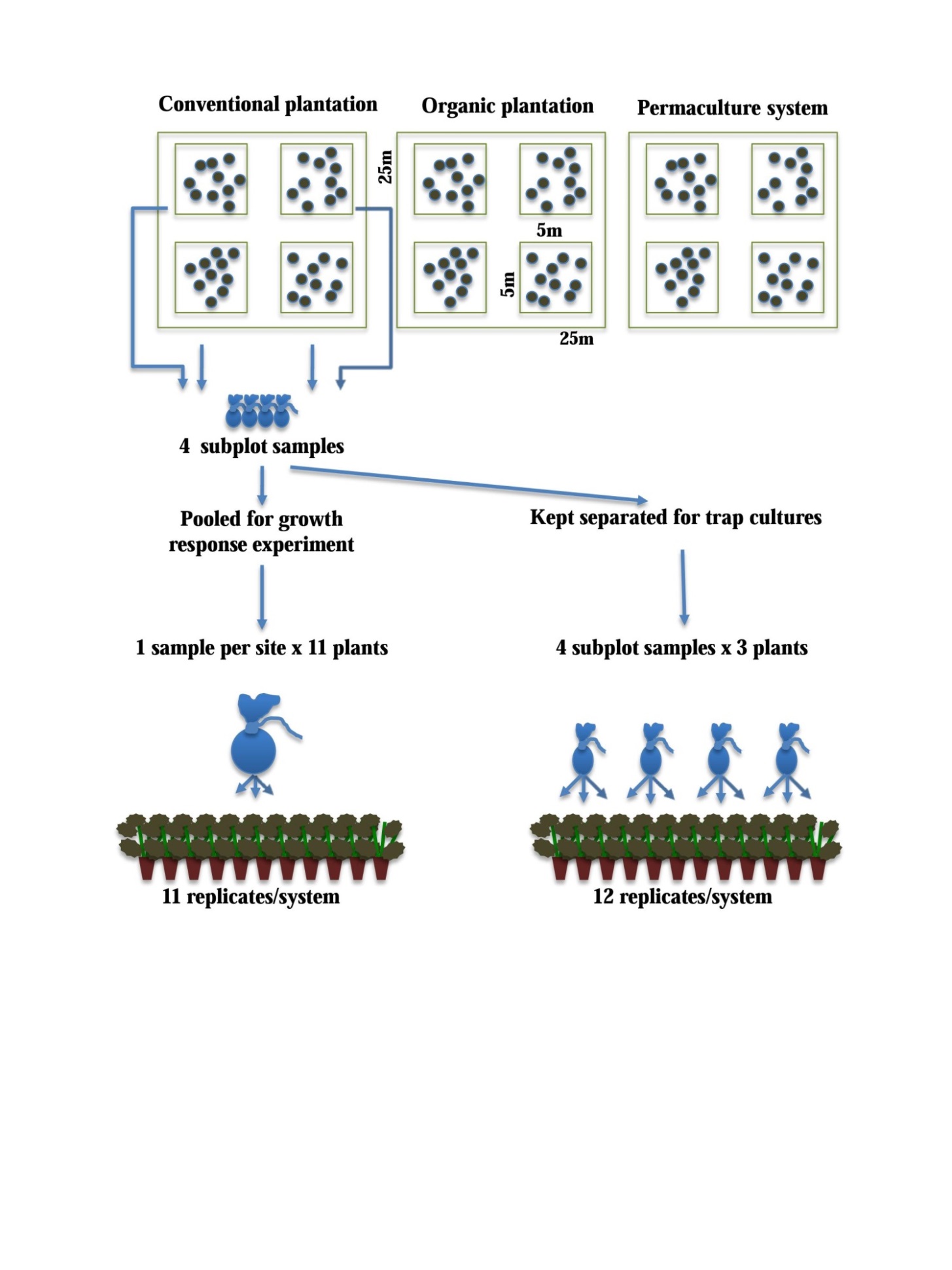


Supplementary Figure S1 Overview scheme of soil sampling and experimental set up. Soil sampling was performed at three differentially managed naranjilla plantation in Guamani (province Napo, Ecuador) within an area of 25 m x 25 m. In each of the three plantation four subplots of 5 m x 5 m were selected to collect a minimum of ten soil subsample. The ten subsamples of each subplot were pooled to get four individual subplot samples per cultivation system. In the growth response experiment, all four subplot samples were pooled to obtain one soil inoculant per system, which was used to inoculate eleven plants. For the trap cultures experiment, every subplot sample was used individually to inoculate three plants yielding a total of twelve replicates per system.


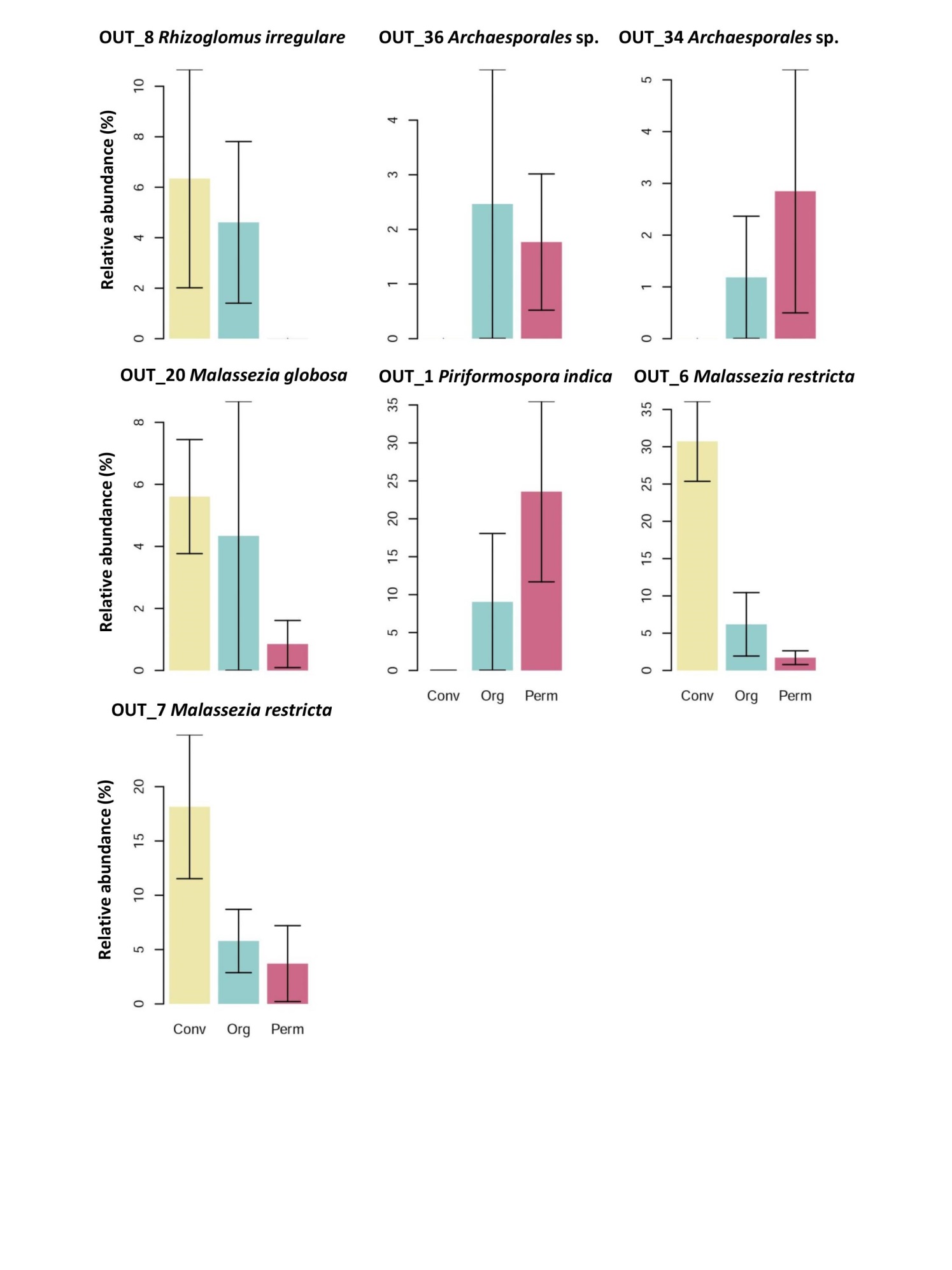


Supplementary Figure S2 Root fungi profiles of ‘abundant operational taxonomic units’ (OUTs) with a minimal relative abundance of 1 % trapped in naranjilla roots supplemented with soil of a conventionally (Conv) and organically (Org) managed naranjilla plantations or from a permaculture (Perm) system.

## Supplementary Tables

Table S1 Detailed overview of analysis parameters of the three local soils collected from differentially managed naranjilla plantation in Guamani (province Napo, Ecuador) used as local soil inoculants, soil collected from two years old naranjilla plantation and the adjacent cloud forest, the experimental substrate and the AMF inocula.

|  |  | Soil / substrate | | | | | | |
| --- | --- | --- | --- | --- | --- | --- | --- | --- |
| **Parameter** | **Unit** | **Conv soil** | **Org soil** | **Perm soil** | **Cloud Forest** | **Naranjilla plantation** | **Experimental substrate** | **AMF inocula** |
| Humus | % G/G | 9.72 | 15.64 | 20.29 | 51.19 | 17.31 | 2.11 | 0.15 |
| Clay | % G/G | 4.78 | 15.32 | 13.94 | 22.24 | 13.38 | 13.48 | 12.88 |
| Silt | % G/G | 64.28 | 50.98 | 46.66 | 17.38 | 36.84 | 16.42 | 14.18 |
| pH-Wert | - | 5.43 | 5.37 | 5.96 | 4.37 | 5.53 | 6.54 | 6.73 |
| Phosphorrus^1^ | mg/kg | 3.6 | 3.2 | 4.7 | 43.6 | 6.7 | 8.7 | 538.3 |
| Potassium^1^ | mg/kg | 43.7 | 115 | 195.2 | 430 | 307.2 | 83 | 665.5 |
| Magnesium^1^ | mg/kg | 17.4 | 52.6 | 192.3 | 599.5 | 177.8 | 481.6 | 1554 |
| Calcium^1^ | mg/kg | 227 | 383 | 1626 | 5109 | 2276 | 2800 | 4371 |
| Salt^2^ | mg/kg | 4.353 | 7.088 | 7.206 | 10.971 | 11.382 | 3.529 | 9.147 |
| P-Olsen | mg/kg | 38.1 | 45.7 | 47 | 75.4 | 63.1 | 22.8 | 20 |
| Ntot | g/kg | 6.9 | 9.6 | 12.3 | 20.4 | 9.7 | 1.9 | 0.6 |
| Nmin | mg/kg | 72.4 | 82.3 | 176.8 | 448 | 262.9 | 98.2 | 10.2 |
| ^1^1:10 EDTA-extraction ^2^H2O5-Ex-lbu  Conv soil = conventionally managed soil, Org soil = organically managed soil, Perm soil = soil from a permaculture system. Soil parameters were analysed by lbu (Labor für Boden- und Umweltanalytik, Eric Schweizer AG, Thun). | | | | | | | | |

Table S2 Shoot phosphorus and nitrogen concentrations of naranjilla plants inoculated with different species of arbuscular mycorrhizal fungi (AMF), local soil inoculants from differentially managed Ecuadorian naranjilla plantations or two control treatments and grown for 14 and 22 weeks in a sterilized potting substrate.

| Treatment | Phosphorus concentration (mg P/g plant) | | | | Nitrogen concentration (mg N/g plant) | | | |
| --- | --- | --- | --- | --- | --- | --- | --- | --- |
|  | Week 14 | | Week 22 | | Week 14 | | Week 22 | |
|  | Mean | SE | Mean | SE | Mean | SE | Mean | SE |
| *Rhizoglomus irregulare* | 3.44 | 0.30 | 2.90 | 0.20 | 13.77 | 1.13 | 15.71 | 0.77 |
| *Claroideoglomus claroideum* | 2.77 | 0.17 | 2.84 | 0.24 | 13.71 | 1.27 | 15.46 | 0.68 |
| *Cetraspora helvetica* | 1.89 | 0.10 | 1.43 | 0.11 | 15.32 | 1.35 | 17.96 | 0.41 |
| AMF mix | 3.41 | 0.08 | 3.18 | 0.12 | 15.99 | 1.46 | 16.17 | 0.68 |
| AMF control | 1.92 | 0.13 | 1.40 | 0.12 | 14.28 | 0.66 | 16.91 | 0.98 |
| Conventional soil | 1.62 | 0.25 | 1.79 | 0.25 | 14.73 | 0.87 | 17.06 | 0.92 |
| Organic soil | 1.94 | 0.17 | 1.86 | 0.27 | 15.93 | 1.69 | 16.45 | 0.64 |
| Permaculture soil | 2.07 | 0.29 | 2.69 | 0.21 | 15.12 | 0.25 | 14.40 | 0.65 |
| Soil control | 1.67 | 0.13 | 1.33 | 0.02 | 13.83 | 0.67 | 15.49 | 0.87 |
| F_ANOVA_ | 14.06 | | 15.39 | | 0.66 | | 2.14 | |
| p-value | < 0.0001 | | < 0.0001 | | ns | | ns | |

SE, standard error; Ns, not significant; AMF mix, mix of the three AMF species *R. irregulare*, *Cl. claroideoglomus* and *Ce. helvetica*; AMF control, sterilized inocula of the three AMF species; soil control, sterilized soil from all three naranjilla plantations. Data represent means (n=5-6); F_ANOVA_ and p-values are also given (ANOVA, Tukey’s honest significant difference (HSD) test, α=0.05).

Table S3 Permutational multivariate analysis of variance (PERMANOVA) of fungal communities in three soils collected from differentially managed naranjilla plantation in Guamani (province Napo, Ecuador).

|  | Degrees of freedom | Sum of squares | Mean squares | F model | R^2^ | Pr(>F) |
| --- | --- | --- | --- | --- | --- | --- |
| Soil | 2 | 1.8662 | 0.93312 | 2.4842 | 0.1507 | 0.002 |
| Residuals | 28 | 10.5175 | 0.37562 |  | 0.8493 |  |
| Total | 30 | 12.3837 |  |  | 1 |  |
